# Prevalence of multiple morbidities and cancers in individuals with Down syndrome: A matched descriptive study using linked electronic health record data

**DOI:** 10.1371/journal.pone.0349794

**Published:** 2026-06-03

**Authors:** Caoimhe McKenna, Kabir Yoshinori Khanna, Meghan A. Cupp, Darlington David Faijue, Anne G.M. Schilder, Arturo Gonzalez-Izquierdo, Muhammad Qummer ul Arfeen, Andre Strydom, Dougal Hargreaves, Rachel Xue Ning Lee, Monica Lakhanpaul, Logan Manikam

**Affiliations:** 1 Great Ormond Street Institute of Child Health, University College London, London, United Kingdom; 2 NI Regional Molecular Diagnostics Service, Belfast Health and Social Care Trust, Belfast, United Kingdom; 3 Queen’s University Belfast, Belfast, United Kingdom; 4 Frimley Health NHS Foundation Trust, Frimley, United Kingdom; 5 Aceso Global Health Consultants Pte Limited, Singapore, Singapore; 6 CAUSALab, Harvard T.H. Chan School of Public Health, Boston, Massachusetts, United States of America; 7 Institute for Infection and Immunity, City St George’s University of London, London, United Kingdom; 8 evidENT, UCL Ear Institute and NIHR UCLH Biomedical Research Centre, London, United Kingdom; 9 Institute of Health Informatics, University College London, London, United Kingdom; 10 Health Data Research UK, London, United Kingdom; 11 Centre for Health Data Science, Department of Applied Health Research, University of Birmingham, Birmingham, United Kingdom; 12 National Institute for Health and Care Research (NIHR) Birmingham Biomedical Research Centre, Birmingham, United Kingdom; 13 Institute of Psychiatry, Psychology and Neuroscience, King’s College London, London, United Kingdom; 14 School of Public Health, Imperial College London, London, United Kingdom; 15 Royal Derby Hospital, University Hospital of Derby and Burton NHS Foundation Trust, United Kingdom; 16 Nottingham University NHS Trust, Nottingham, United Kingdom; 17 Department of Epidemiology and Public Health, Institute of Epidemiology and Health Care, University College London, United Kingdom; 18 Saw Swee Hock School of Public Health, National University of Singapore, Singapore, Singapore; 19 The Jockey Club School of Public Health and Primary Care, Faculty of Medicine, The Chinese University of Hong Kong, Hong Kong SAR, China; University of Oxford, UNITED KINGDOM OF GREAT BRITAIN AND NORTHERN IRELAND

## Abstract

**Background:**

Down syndrome is associated with the development of multiple morbidities throughout the life course, yet comprehensive data on its relative burden remains limited. This descriptive, matched, retrospective cohort study aimed to assess the 20-year period prevalence of morbidities and cancers in adults and children with Down syndrome.

**Methods:**

We analysed electronic health record data from January 1998 to December 2017, matching individuals with Down syndrome to up to five matched controls. Period prevalence and odds ratios (OR) were calculated for 30 morbidities and 24 cancers.

**Results:**

This study included 4,648 individuals with Down syndrome (32,920 person-years) and 23,238 matched controls (236,883 person-years). Most morbidities had a significantly higher period prevalence in individuals with Down syndrome, including hypothyroidism (30.4%), congenital cardiac disease (27.8%), and epilepsy (21.9%). We found an increased comparative risk of autism (OR 7.5, 95% CI 6.4–8.0), chronic kidney disease (OR 2.4, 95% CI 2.1–2.8) and inflammatory bowel disease (OR 2.5, 95% CI 2.2–2.8). Individuals with Down syndrome also had a significantly higher period prevalence of leukaemia and testicular cancer. Conversely, most solid tumours were less prevalent in individuals with Down syndrome.

**Conclusions:**

This study presents findings from one of the largest described cohorts of individuals with Down syndrome, contributing to an understanding of the comparative prevalence of multiple comorbidities and cancers among both adults and children with Down syndrome. These findings support prioritising surveillance for a range of conditions such as hypothyroidism and childhood leukaemia and may justify de-emphasising routine screening for several solid tumours in Down syndrome.

## Introduction

Down syndrome (DS) is associated with the development of multiple health conditions throughout the life course, affecting nearly every system in the body. These include congenital heart defects [1,2], leukaemia [3,4], disorders of the thyroid [5], impairment of vision [6] and hearing [7], disorders of the gastrointestinal tract [8], immune deficiency [9], respiratory tract infections [10], sleep disordered breathing [11], instability of the cervical spine [12,13], dementia [14,15] and various malignancies [16,17]. However, no large UK EHR study has simultaneously estimated period prevalence across a broad panel of morbidities and cancers in both adults and children with DS. Our study addresses this gap.

Although the increased risk of leukaemia among individuals with DS is well-established [16], there is ongoing debate about the risks of solid tumours in individuals with DS. While some studies have indicated that solid tumours are less frequently diagnosed in individuals with DS [18], others report greater than expected rates of testicular cancer among men with DS [17,19] and increased rates of liver [20], gastric [21], and ovarian cancer [22], as well as retinoblastoma [21] and lymphoma [19]. There are few contemporary findings that provide strong evidence of increased solid cancer rates. The ongoing lack of clarity about the relative burdens of malignancies in DS is partially attributable to the limited number of large-scale observational studies investigating the occurrence of multiple tumour types among individuals with DS.

Much of the existing literature on the prevalence of DS-associated morbidities and cancers in individuals with DS is based on retrospective chart reviews and has small sample sizes. Due to these limitations, the reported figures vary considerably. Furthermore, very few of these studies include an appropriate comparator group of individuals who do not have DS, preventing meaningful relative comparisons. Additionally, these studies tend to focus on the adult population, limiting their generalisability to children with DS.

Obtaining more precise estimates of the prevalence of morbidities and cancers is key to understanding the burden of disease among individuals living with DS. Such awareness can inform clinical decisions and health surveillance practices: for example, active screening for conditions that are common in DS and reduced screening for those conditions which are rare. This is of particular relevance to paediatric surveillance, as it can guide appropriately timed screening and intervention practices. An accurate estimation of disease prevalence also informs allocation of health resources to best serve patient needs. Finally, understanding the burden of disease in the DS population informs future research and highlights health conditions which require increased academic attention to advance understanding of how DS may influence their aetiology.

In this study we utilised a large linked dataset (CPRD-HES-NCRAS-ONS) to determine the prevalence of a multitude of DS-associated morbidities and cancer in adults and children in a large cohort of individuals with DS. Our study explores cancer prevalence in individuals living with DS, aiming to provide greater clarity in an area where existing research has produced inconsistent findings. Additionally, we stratified prevalence into adult and childhood period prevalence to assess the morbidities and cancers with an earlier onset in the individual’s lifespan.

## Methods

This descriptive, matched, retrospective cohort study utilised population-based, linked electronic health record (EHR) data in England. This data is provided by the Clinical Practice Research Datalink (CPRD) and is linked to Hospital Episode Statistics (HES), The National Cancer Registration and Analysis Service (NCRAS) and Office for National Statistics (ONS) datasets. Further descriptions of these datasets can be found in S1 Appendix.

This study was carried out with the support of the ClinicAl Disease Research Using Linked Bespoke Studies and Electronic Health Records (CALIBER©) resource. CALIBER, by the University College London (UCL) Institute of Health Informatics, provides validated EHR phenotyping algorithms and other tools for national structured data sources.

### Data sources

The CPRD dataset is a longitudinal primary care database of anonymised EHRs from general practitioners (GPs). CPRD is a rich source of patient data providing information on demographics, symptoms, health behaviours, diagnoses, investigations, referrals, procedures, vaccinations, and prescriptions [23] and commonly used in population studies [24]. The patient-level data, drawn from a subset of primary care practices based in England, is de-identified and linked to secondary care (HES) [25], the NCRAS [26], and mortality and deprivation data (ONS). The use of multiple linked data sources provides complementary and corroborating longitudinal information about an individual’s medical history. The linkage utilised in this study (linkage 16) includes data from 264 practices, covering 3.46% of the UK.

Within the participating practices, 88% of eligible patients have the necessary data to allow linkage to HES, Cancer Registry, and the ONS Death Registration data. Within the participating practices, 97% of acceptable patients are eligible to be linked to data on the IMD and Townsend socioeconomic scores.

Data in CPRD is recorded using Read codes, which map to Systematic Nomenclature of Medicine – Clinical Terms [27,28]. These read codes can be found in S1 Table. Data entry and completeness in recording is also enhanced by the Quality and Outcomes Framework [25,29] which provides incentive payments for GPs to record key data items (e.g., smoking status) and the delivery of services to key patient groups*.*

Validation of CPRD data has shown high positive predictive value and comparable disease incidences with other UK data sources [30–33]. A systematic review of these CPRD validation studies also found that diagnoses were generally dependable [34]. Studies have also shown that CPRD patients are broadly representative of the UK population in terms of age, sex, ethnicity, body mass index (BMI) and mortality [23,35,36] and the dataset is used widely for epidemiological research [23].

### Study population

The CPRD subset for this study included the CPRD GOLD database eligible for linkage to NCRAS, HES, and ONS data from January 1998 to June 2018. In June 2018, it had coverage of approximately 15 million patients from 735 practices in the UK [26]. This subset included the anonymised electronic health care records for individuals with DS and their matched controls. Access to CPRD data was granted on 07/06/2017. None of the authors had access to information that could identify individual participants during or after data collection.

The eligibility for the cohort (herein referred to as ‘start of follow-up’) began on the latest of the individual’s registration date, the practice Up To Standard (UTS) date (deemed as the date at which the data provided by the practice has continuous high-quality data), and 01/01/1998. Individuals with DS were defined as registered patients with at least one Down syndrome diagnosis code at any time, in their primary care (CPRD) or hospital care (HES) records. Females with a first record of DS after pregnancy were excluded, as they may represent the mothers of children with DS, as opposed to individuals with DS. They were also ineligible to act as a control. The control group consisted of registered patients with no record of DS diagnosis in their primary or secondary care records. Individuals with DS were matched with up to five matched controls based on GP practice, practice level IMD, year of birth ± 1 year, sex and index date (the date at which a case is first labelled as having DS).

Participants’ exit from the cohort (herein referred to as ‘end of follow-up’) was defined as the earliest of the individual’s transfer out date, the practice last collection date, date of death (defined as the ONS date of death, or where missing, the ‘CPRD derived date of death’), and 31/12/2017. Last Observation Carried Forward and data linkage was used to minimise the impact of missing data.

### Outcomes

#### DS-associated morbidities and cancers.

The occurrence of a DS-associated morbidity or cancer was defined as a positive record of that condition within the dataset. Cancer diagnoses were defined according to body site, and their categorisation reflects that used in existing epidemiological studies [20,37]. The full list of 30 morbidities and 24 cancers investigated in this study is found in S2 Table.

The phenotyping code lists were developed in a stepwise process: (1) Compiling code lists utilised and published in existing peer-reviewed research, examining the same conditions of interest; (2) Searching the Read and ICD-10 complete code database using relevant terms, and (3) Reaching expert consensus on which codes should be included to define the condition of interest.

The final phenotyping code lists for each condition were compiled by three academic clinicians and two researchers with experience in the field of epidemiology and research using electronic health records.

### Statistical analysis

We estimated the 20-year period prevalence of DS-associated morbidities and cancers as the number of individuals with any record of the condition of interest, as defined by the phenotyping code lists, over the total number of eligible individuals.

We used Chi-squared and Fisher’s Exact test for categorical outcomes and two sample t-tests and Mann-Whitney U-test for continuous outcomes to compare the outcomes in those with DS and the matched controls. A p-value threshold of <0.01 was used to determine statistical significance [38] and 95% CIs were also calculated for all measures of average and proportions.

Unadjusted odds ratios (OR) were calculated for the occurrence of DS-associated morbidities and cancers in those with DS versus matched controls using logistic regression. We prioritised unadjusted ORs to describe the overall burden in DS given matched controls, in keeping with best practice in descriptive research, as per Lesko, Fox & Edwards [39]. We also conducted sensitivity analyses adjusting for ethnicity, smoking and person-years to reflect the controlled direct effect of DS on the conditions of interest, within levels of these covariates.

In the primary analysis, outcomes were determined for the entire study population (i.e., including children and adults). We present a subgroup analysis for children, defined as those aged <18 years at the end of follow-up, with a focus on the primary outcomes common in childhood: leukaemia, lymphoma, and neuroblastoma [37]. Further subgroup analysis included the population aged ≥40 years and ≥30 years at the beginning of follow-up and focused on ischaemic heart disease and dementia, due to the adult-onset nature of these conditions and existing literature on the age of disease onset [40–43].

### Primary outcomes

1. The 20-year period prevalence of DS-associated morbidities among children and adults with DS, and a comparison with matched controls.
2. The 20-year period prevalence of cancers among children and adults with DS, and a comparison with matched controls.

### Secondary outcomes

1. Odds of occurrence of DS-associated morbidities and cancers in individuals with DS, compared to matched controls.

### Ethical approval

Approval for access to the dataset was granted by the MHRA (UK) Independent Scientific Advisory Committee (ISAC), under Section 251 (NHS Social Care Act 2006), in 2018 (protocol number: 17_009R). The ISAC is a non-statutory expert advisory body established in 2006 by the Secretary of State to provide advice on research related requests to access data provided by CPRD.

Access to the database was via the UCL Data Safe Haven, which requires data to be stored and analysed within a secure platform. Data users are provided with pseudo-anonymised data only and are obliged to comply with confidentiality standards.

### Governance

Research Design approval was sought and obtained from the Joint Research and Development Office at the Great Ormond Street Institute of Child Health, UCL (R&D number 17PP09).

## Results

### Demographics of the DS cohort and their matched controls

There were 4,648 individuals with DS and 23,238 matched controls in the eligible cohort. In the paediatric subgroup, data was available for 1,340 children with DS and 6,711 matched controls, who were aged ≤18 years at the end of follow-up.

In the primary analysis, individuals with DS contributed on average 5.7 years of follow-up and matched controls contributed 10.6 years. Individuals with DS had greater mortality during the study period, compared to matched controls (24.1% [95% CI 22.8%−25.3%] v. 3.4% [95% CI 3.2%−3.7%], p < 0.001).

The predominant recorded ethnicity of individuals in both the DS cohort and the matched controls was “White.” The mean Body Mass Index (BMI) of individuals with DS was significantly higher than that of matched controls (29.3 kg/m^2^ [95% CI 29.0–29.5 kg/m^2^] v. 26.3 kg/m^2^ [95% CI 26.3–26.4 kg/m^2^] respectively, p < 0.001). Individuals with DS were significantly less likely to have any record of smoking (9.1% [95% CI 8.2%−10.2%] v. 48.9% [95% CI 48.1%−49.7%] of matched controls, p < 0.001).

In the subgroup analysis of children only, children with DS contributed on average 3.9 years of follow-up and matched controls contributed 6.1 years. Children with DS had greater mortality during the study period, compared to matched controls (3.1% [95% CI 2.3%−4.2%] v. 0.3% [95% CI 0.2%−0.4%], p < 0.001).

The mean BMI of children with DS was significantly higher compared to matched controls, 27.1 kg/m^2^ (95% CI 25.7–28.4 kg/m^2^) v. 22.3 kg/m^2^ (95% CI 21.7–23.0 kg/m^2^) (p < 0.001) respectively. Table 1 summarises key demographic characteristics of the DS cohort and controls.

**Table 1:** Summarising and comparing the demographic characteristics of the Down syndrome (DS) cohort and the matched control group, in the primary & subgroup analysis.

	Primary analysis (adults & children)			Subgroup analysis (≤18yrs at end of follow-up)		
	DS Cohort N = 4,648 *n* (%)/average (95% CI)	Matched Control Group N = 23,238 *n* (%)/average (95% CI)	p-value* (p < 0.01)	DS cohort, children only N = 1,340 *n* (%)/average (95% CI)	Matched control group, children only N = 6,711 *n* (%)/average (95% CI)	p-value* (p < 0.01)
**PERSON-YEARS**						
Total years contributed	32,919.8	236,883.0		6,889.9	45,464.3	
Median years contributed per person	5.7yrs (5.4–6.0yrs)	10.6yrs (10.6–10.7yrs)	**<0.001’**	3.9yrs (3.6–4.2yrs)	6.1yrs (5.9–6.3yrs)	**<0.001’**
**GENDER**						
Male	2,551 (54.0%)	12,553 (54.0%)	0.996	698 (52.1%)	3,443 (51.3%)	0.599
**Age**						
Median age at start of follow-up (yrs):	26yrs (25–28) Range: 0–74yrs	25yrs (24–26) Range: 0–72yrs	0.022’	1yr (1–2yrs) Range: 0–18yrs	0yrs (0−0yrs) Range: 0–18yrs	**<0.001’**
Median age at end of follow-up (yrs):	35yrs (33–36) Range: 0–75yrs	33yrs (33–34) Range: 0–88yrs	0.090’	8yrs (8–9yrs) Range: 0–18yrs	9yrs (8–9yrs) Range: 0–18yrs	0.377‡
**DATA ENTRY**						
**Age range at start of follow-up^**						
0-5 years	1,013 (21.8%)	6,011 (25.9%)	**<0.001**	958 (71.5%)	5,593 (83.3%)	**<0.001**
6-10 years	352 (7.6%)	1,718 (7.4%)	0.669	229 (17.1%)	739 (11.0%)	**<0.001**
11-18 years	475 (10.2%)	2,319 (10.0%)	0.619	153 (5.7%)	379 (11.4%)	**<0.001**
19-30 years	686 (14.8%)	3,099 (13.3%)	0.010	–	–	–
31-60 years	1,931 (41.5%)	9,589 (41.3%)	0.723	–	–	–
>60 years	191 (4.1%)	502 (2.2%)	**<0.001**	–	–	–
**Age range at end of follow-up^**						
0-5 years	449 (9.7%)	2,241 (9.6%)	0.972	449 (33.5%)	2,241 (33.4%)	0.935
6-10 years	385 (8.3%)	1,895 (8.2%)	0.771	385 (28.7%)	1,895 (28.2%)	0.714
11-18 years	506 (10.9%)	2,575 (11.1%)	0.699	506 (37.8%)	2,575 (38.4%)	0.676
19-30 years	764 (16.4%)	4,173 (18.0%)	0.013	–	–	–
31-60 years	2,092 (45.0%)	8,906 (38.3%)	**<0.001**	–	–	–
>60 years	452 (9.7%)	3,448 (14.8%)	**<0.001**	–	–	–
**Total person-years contributed per age group** (at start of follow-up)						
0-5 years	6,566.8 (19.9%)	47,505.4 (20.1%)	0.671	5,617.4 (81.5%)	40,215.9 (88.5%)	**<0.001**
6-10 years	2,804.6 (8.5%)	19,119.2 (8.1%)	**0.005**	926.8 (13.5%)	4,230.3(9.3%)	**<0.001**
11-18 years	3,769.3 (11.5%)	24,228.4 (10.2%)	**<0.001**	345.7 (5.0%)	1,018.1(2.2%)	**<0.001**
19-30 years	5,467.8 (16.6%)	27,870.7 (11.8%)	**<0.001**	–	–	–
31-60 years	13,811.0 (41.9%)	113,153.5 (47.8%)	**<0.001**	–	–	–
>60 years	500.3 (1.5%)	5,005.9 (2.11%)	**<0.001**	–	–	–
**DATA EXIT**						
Death during follow-up	1,119 (24.1%)	796 (3.4%)	**<0.001**	42 (3.1%)	17 (0.3%)	**<0.001**
**ETHNICITY**						
White	3,510 (85.7%)	14,440 (84.11%)	0.013	990 (77.8%)	4253 (77.2%)	0.652
Bangladeshi/ Pakistani/ Indian/ Chinese/ Asian other	138 (3.4%)	666 (3.9%)	0.124	84 (6.6%)	345 (6.3%)	0.656
Black African /Caribbean /Other	123 (3.0%)	413 (2.4%)	0.029	87 (6.8%)	218 (4.0%)	**<0.001**
Mixed /Other	326 (8.0%)	1,651 (9.6%)	**0.001**	111 (8.7%)	218 (4.0%)	**<0.001**
**SOCIOECONOMIC STATUS** *(Practice Level Index of Multiple Deprivation)*						
1 (highest)	596 (12.8%)	2,980 (12.8%)	0.998	181 (13.5%)	923 (13.8%)	0.811
2	849 (18.3%)	4,243 (18.3%)	0.991	246 (18.4%)	1,239 (18.5%)	0.929
3	965 (20.8%)	4,825 (20.8%)	0.998	253 (18.9%)	1,293(19.3%)	0.743
4	1,096 (23.6%)	5,480 (23.6%)	0.998	329 (24.6%)	1,593(23.7%)	0.523
5 (lowest)	1,142 (24.6%)	5,710 (24.6%)	0.998	331 (24.7%)	1,663(24.8%)	0.951
**AVERAGE BODY MASS INDEX (BMI) kg/m2**						
Mean BMI (kg/m^2^)	29.29 (29.0-29.5)	26.34 (26.3-26.4)	**<0.001†**	27.1 (25.7-28.4)	22.3 (21.7-23.0)	**<0.001†**
**SMOKING STATUS**						
Non-smoker	2,899 (90.9%)	8,097 (51.1%)	**<0.001**	–	–	–
Smoker (any history)	291 (9.1%)	7,752 (48.9%)	**<0.001**	–	–	–
**GEOGRAPHICAL REGION**						
1	81 (1.7%)	405 (1.7%)	0.999	20 (1.5%)	94 (1.4%)	0.795
2	695 (14.9%)	3,475 (14.9%)	0.998	174 (13.0%)	854 (12.7%)	0.795
3	173 (3.7%)	865 (3.7%)	0.999	65 (4.9%)	320 (4.8%)	0.897
4	145 (3.1%)	725 (3.1%)	0.999	40 (3.0%)	218 (3.3%)	0.617
5	490 (10.5%)	2,448 (10.5%)	0.988	139 (10.4%)	678 (10.1%)	0.765
6	433 (9.3%)	2,165 (9.3%)	0.999	121 (9.0%)	630 (9.4%)	0.681
7	684 (14.7%)	3,420 (14.7%)	0.998	182 (13.6%)	915 (13.6%)	0.959
8	585 (12.6%)	2,925 (12.6%)	0.998	164 (12.2%)	836 (12.5%)	0.825
9	794 (17.1%)	3,970 (17.1%)	0.998	281 (21.0%)	1379 (20.6%)	0.728
10	568 (12.2%)	2,840 (12.2%)	0.998	154 (11.5%)	787 (11.7%)	0.807

Nb. Cases (individuals with DS) are matched with at least 4 matched controls (non-DS individuals) based on GP practice, practice level index of multiple deprivation, year of birth ± 1 year, sex, and index date.

*p Values calculated using χ2 (comparison of proportions)

‡p Values calculated using Fisher’s exact test (comparison of proportions, non-parametric)

†p Values calculated using t-test (comparison of normally distributed continuous variables)

‘p Values calculated using Mann-Whitney U-test (comparison of non-normal continuous variables)

Missing data: Primary analysis: Gender = 0, Ethnicity: DS = 551, Control = 6,068; SES:0; BMI DS = 1,989, Control = 10,285; Smoking status: DS = 1,458 Controls = 7,389; Geographical region = 0; Subgroup analysis: Gender = 0, Ethnicity: DS = 68, Control = 1,205; SES:0; BMI DS = 1,258, Control = 6,487; Geographical region = 0

^Start of follow-up is defined as the latest of the patient registration date, the practice UTS date, and 01/01/1998. End of follow-up is defined as the earliest of the patient transfer out date, the practice last collection date, date of death and 31/12/2017.

Yrs: Years.

### The study period prevalence and odds of DS-associated morbidities in the DS cohort and controls

The morbidities among individuals with DS with the highest prevalence during our observation period were hypothyroidism (30.4%), eczema (29.1%), congenital cardiac disease (27.8%), epilepsy (21.9%), hearing impairment (19.2%) and dementia (17.6%). For 25 of the 33 morbidity outcomes examined, the study period prevalence was significantly higher among individuals with DS, compared with matched controls. A further three morbidities were close to reaching statistical significance (iron deficiency anaemia, ischaemic heart disease, schizophrenia). Of all the morbidities examined, only two were significantly *less* likely than the matched controls to have a positive record: anxiety/depression (14.1% (95% CI 13.2%−15.2%) v. 21.4% (20.9%−21.9%), p < 0.001) and ischaemic heart disease in those aged over 40 years at the start of follow-up (9.6% (95% CI 8.2%−11.2%) v. 13.8% (95% CI 13.0%−14.7%), p < 0.001). The biggest differences in the 20-year period prevalence of morbidities between the DS cohort and matched controls were in dementia amongst those aged over 30 years at the start of follow-up (37.3% v. 1.5%), hypothyroidism (30.4% v. 3.2%), congenital cardiac disease (27.8% v. 0.9%), epilepsy (21.9% v. 2.6%) and hearing impairment (19.2% v. 3.7%).

**Fig 1:**
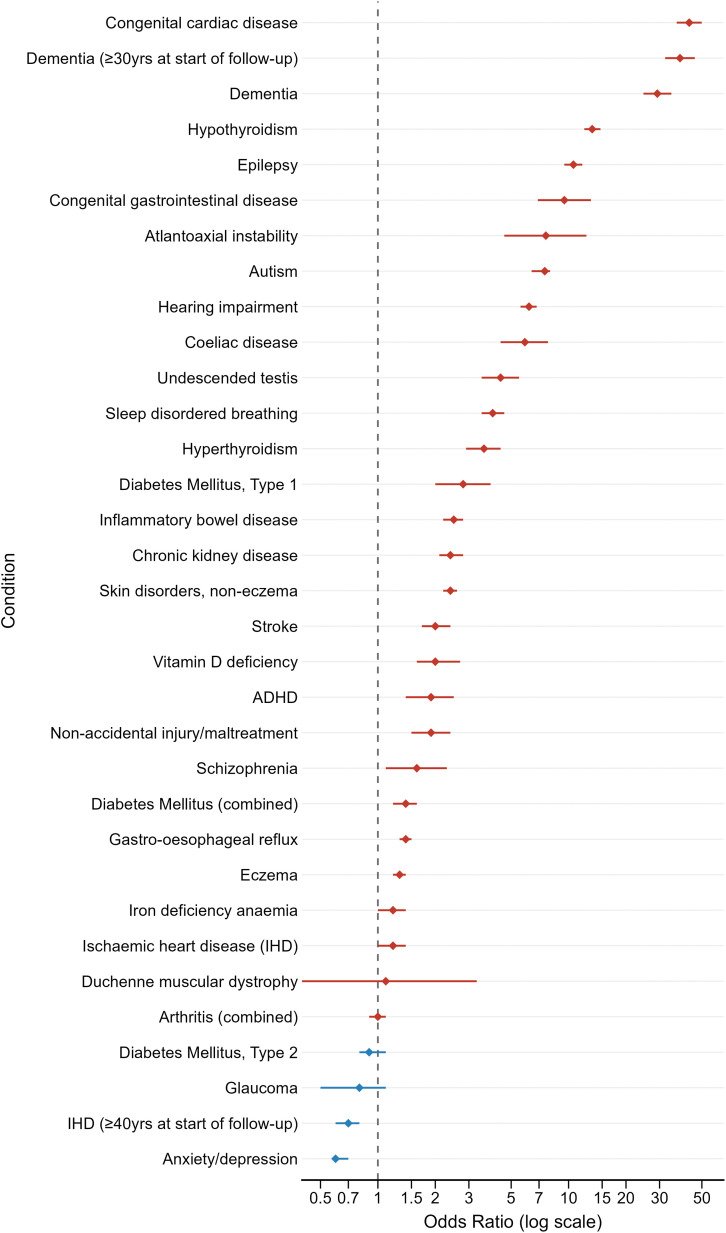
Odds ratios (95% CI) of morbidities in individuals in the DS cohort v. the matched control group. **In individuals aged >30 years at the start of follow-up.

Fig 1 presents the odds ratios (OR) for the 32 morbidity outcomes examined in the DS cohort versus matched controls. The ORs mirror the differences in disease prevalence, with most morbidities having a significantly increased OR. The largest relative differences were for congenital heart disease, dementia in individuals over 30 years at the start of follow-up, epilepsy, hearing impairment and hypothyroidism. S3 Table displays the full period prevalences and odds ratios for each morbidity.

### The study period prevalence and odds of cancers in the DS cohort and matched controls

Two hundred and seventeen individuals with DS (4.7% (95% CI 4.1%−5.3%)) and 2,421 of the matched controls (10.4% (95% CI 10.0%−10.8%)) had a record of any of the 24 cancers examined (p < 0.001). The most prevalent cancers among individuals with DS were leukaemia (1.0%), non-melanomatous skin cancer (0.6%), colorectal (0.5%), testicular (0.4%), breast (0.3%) and uterine cancer (0.3%). The full results are seen in S4 Table.

When comparing the period prevalences of cancers in the DS cohort and the matched controls, individuals with DS were significantly more likely to have a record of leukaemia (1.0% v. 0.2%) and testicular cancer (0.4% v. 0.1%). However, they were significantly less likely to have a record of breast (0.3% v. 1.3%), cervical (0.1% v 1.9%), colorectal (0.5% v. 1.8), lung (0.1% v. 0.5%) melanoma (0.1% v. 0.5%), non-melanomatous skin (0.6% v. 2.3%), prostate (0.1% v. 0.6%) and uterine cancer (0.3% v. 1.3%).

**Fig 2:**
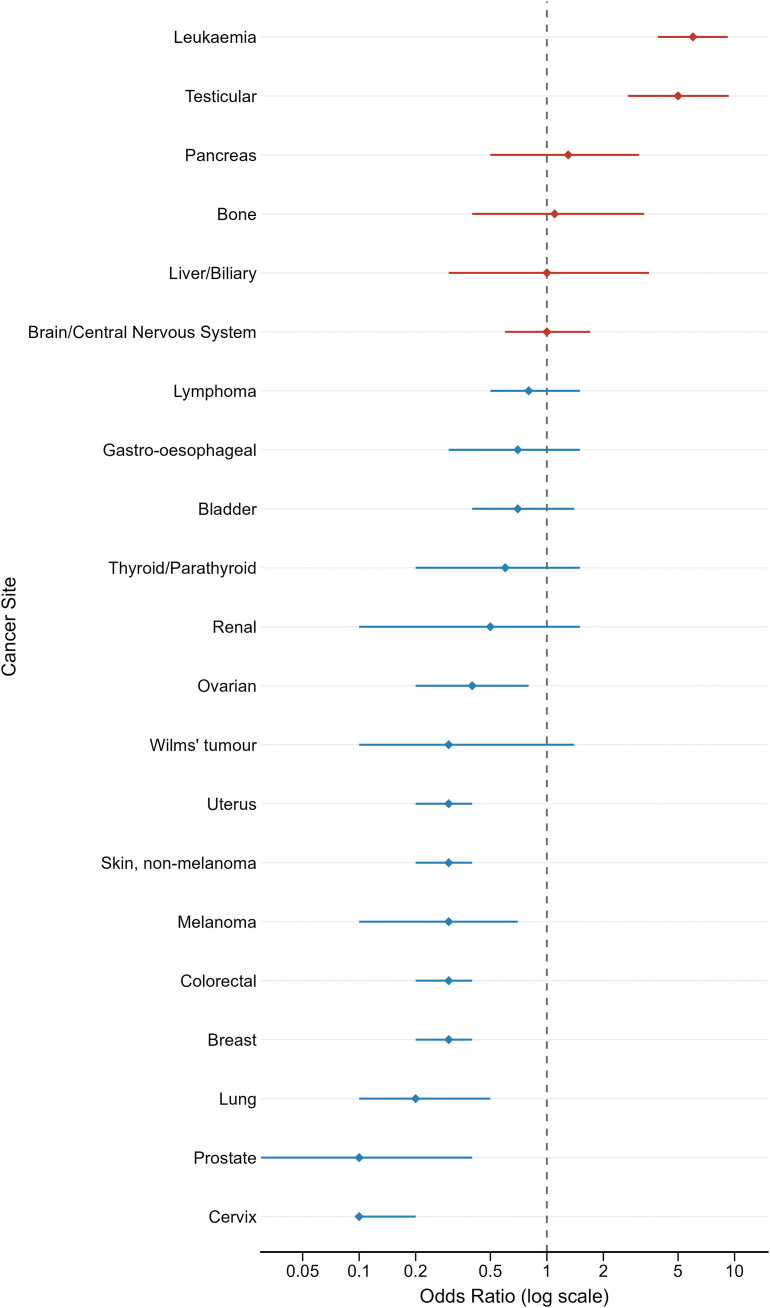
Odds ratios (95% CI) of cancers in individuals in the DS cohort vs. the matched control group.

The odds ratios of each cancer assessed in this study are illustrated in Fig 2.

### Subgroup analysis, DS-associated morbidities in children only

In the subgroup analysis, looking at morbidities and cancers among children only, the most prevalent morbidities among children with DS were congenital heart disease (56.3%), eczema (24.1%), hearing impairment (23.5%), sleep disordered breathing (19.1%) and gastro-oesophageal reflux (19.0%).

Among the 29 morbidities examined in children, those with DS had a significantly higher period prevalence of 23 of the conditions, compared to matched controls. The biggest differences in the period prevalence of morbidities, when comparing children with DS and their matched controls, were in congenital cardiac disease (56.3% v. 1.1%, OR 118.6), hearing impairment (23.5% v. 2.5%, OR 12.2) sleep disordered breathing (19.1% v. 1.6%, OR 15.0), hypothyroidism (15.8% v. 0.5%, OR 39.2) and gastro-oesophageal reflux disease (19.0% v. 4.2%, OR 5.4). However, they were significantly less likely to have a record of eczema (24.1% (95% CI 21.9%−26.5%) v. 31.6% (95% CI 30.5%−32.7%), p < 0.001). The reduced period prevalence of anxiety/depression among children with DS versus matched controls was also close to reaching statistical significance (1.9% (95% CI 1.3%−2.8%) v. 2.8% (95% CI 2.5%−3.2%), p = 0.048), but the absolute difference was small. The results are illustrated in full in S5 Table.

### Subgroup analysis, cancers in children only

Leukaemia was the most prevalent cancer among children with DS (2.2% (95% CI 1.5%−3.1%)) and this was significantly higher when compared with matched controls (0.03% (95% CI 0.01%−0.12%), p < 0.001). The period prevalence of lymphoma and neuroblastoma was not significantly different between children with DS and matched controls.

**Fig 3:**
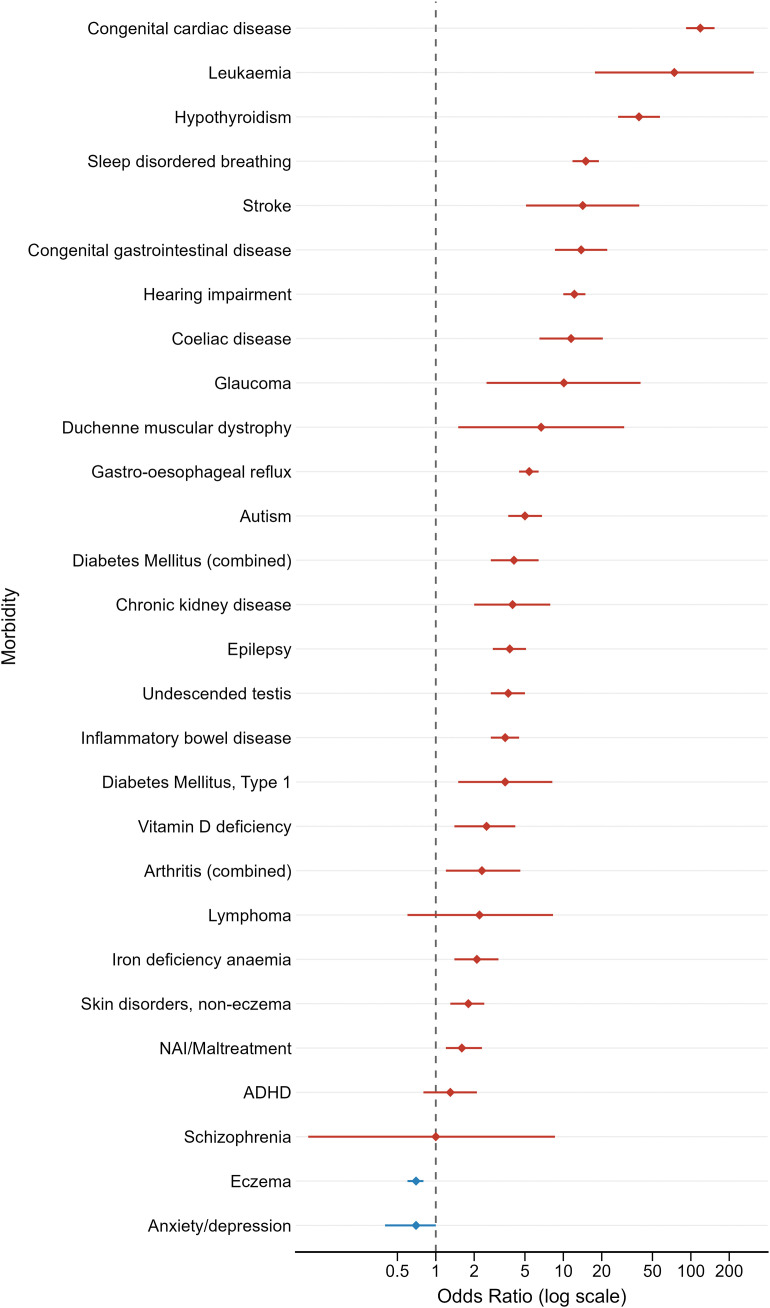
Odds ratios (95% CI) of morbidities and cancers in children only with DS vs. children only in the matched control group.

Fig 3 highlights the odds ratios of the morbidities and cancers in the subgroup analysis.

## Discussion

### Statement of principal findings

Most of the morbidities examined had a higher 20-year period prevalence in individuals with DS, compared with matched controls. The most prevalent conditions among those with DS, and those with the largest difference in prevalence when comparing DS cases and matched controls, reflect conditions which are frequently described in the DS literature, such as hypothyroidism, congenital heart disease, epilepsy, hearing impairment, and dementia.

However, the findings presented here also highlight a significantly increased period prevalence of several additional morbidities that have been poorly documented in existing literature. These include autism, chronic kidney disease, diabetes mellitus type 1, inflammatory bowel disease, non-accidental injury/maltreatment, and vitamin D deficiency. Notably, there were two DS-associated morbidities which appeared to be significantly *less* prevalent in the DS cohort versus matched controls: anxiety/depression and ischaemic heart disease in individuals aged over 40 years.

Regarding cancer, individuals with DS had a higher prevalence of diagnosis with leukaemia and testicular cancer, compared with matched controls. Individuals with DS appeared to have a significantly lower overall period prevalence of the cancers combined. Specifically, they had a lower prevalence of breast, cervical, colorectal, lung, melanoma, non-melanomatous skin cancer, ovarian, prostate and uterine cancers.

The period prevalence of smoking is likely to be exaggerated compared to the wider UK population due to recording bias, as a positive history of smoking is much more likely to be recorded in the health record than “no history” [44]. Both a raised BMI and a lower prevalence of smoking are in keeping with what would be expected in the DS population, compared to the general population [45–47].

### Strengths

#### The size of the linked dataset.

A key strength of the linked dataset utilised in this study is its size. The DS population included in this study represents one of the largest DS cohorts reported in the academic literature to date. This large dataset made it possible to study rare outcomes, including congenital abnormalities and cancers, and increased statistical power to detect differences between populations. Furthermore, our dataset includes both children and adults with DS, allowing us to gain a picture of disease burden across the life course without being limited to a single time period, such as adulthood. In the existing literature examining the occurrence of morbidities in individuals with DS, small sample sizes, absence of a comparator group, and a paucity of data on childhood are recurrent themes.

#### Diagnostic reliability.

The linkage of primary care, secondary care and cancer registry datasets enabled access to broader patient data. Therefore, it is less likely that a diagnosis was omitted from our data, compared with relying on one data source alone. Using data from linked primary/secondary care may build on and exceed clinic cohorts in previous studies, with increased healthcare contact raising ascertainment of morbidities.

However, the analysis was still reliant on the sensitivity and specificity of coding within the dataset. The presence or absence of a disease was defined as the presence or absence of a coded entry in the dataset. Codes are entered by many individuals, predominantly coding technicians, and primary care physicians, some of whom have had no direct contact with the patient. Furthermore, coders may use different codes to define the same clinical terminology. To address this, the phenotyping code lists used in the analysis were extensive and reviewed by clinicians and researchers with experience in the field of epidemiology and research utilising electronic health records. However, it is still possible that some diagnoses were not recorded in the dataset, and that some were misclassified, leading to over- or underestimates in disease prevalence.

The use of such a large dataset, with large participant numbers, is likely to reduce the impact of misclassification. Also, since the introduction of payment by results (PbR) in 2002, financial incentives have been introduced to enhance coding depth. This has resulted in an increase in the number of diagnostic codes used and improvements in coding accuracy [48].

### Linkage accuracy

The use of linked datasets relies on the quality of linkage, using patient identifiers. Within HES, linkage algorithms rely upon the accurate recording of NHS numbers. Hagger-Johnson et al. estimated a “patient identifier mismatch” of 4% in the HES dataset [49].

Errors in data linkage of electronic health records are known to result in significantly different conclusions about the association between exposures and outcomes [50]. However, the linkage methods used by NHS Digital on the CPRD population, for the constituent datasets available through CALIBER have been used in multiple comparable projects and have demonstrated high quality matching [51].

### Limitations

#### The impact of differences in health surveillance on the disease prevalence.

It must also be considered that individuals are more likely to be diagnosed with a condition where there is active screening. In the UK, guidelines from the Royal College of Paediatrics and Child Health (RCPCH) [52], Royal College of General Practitioners (RCGP) [53], and the Down syndrome Medical Interest Group (DSMIG) [54] recommend further health screens to those recommended for individuals without DS. These include assessments of thyroid function, hearing and vision. This may amplify the association between DS and the condition of interest and result in a relative underestimate of disease prevalence in the matched controls. Similarly, for those conditions where screening is not common practice in the DS population, such as sleep disordered breathing and diabetes mellitus, the true disease prevalence may be underestimated. An avenue of potential further exploration of this challenge might involve comparing conditions that are actively surveyed in a population of individual with and without DS.

#### The choice of unadjusted odds ratio.

The choice of logistic regression and to present odds ratio was a modelling decision. Caution must be employed when interpreting ORs in the context of common diseases as ORs may exaggerate the magnitude of significance in these scenarios. Other measures, such as risk ratio (RR), may be more intuitive, but methods to estimate RRs using ORs risk non-convergence [55]. Further, for relatively rare conditions, such as many of those explored in this paper, the magnitude of OR and RR are comparable [56], hence the choice of OR.

The odds ratio was presented unadjusted, in line with existing practices of data presentation in descriptive studies [39]. This allows an illustration of the overall burden of comorbidities in individuals with DS, including those potentially mediated by other factors such as lifestyle factors.

To explore the controlled direct effect of DS, we conducted an analysis adjusting the odds ratio for smoking and ethnicity, as well as time-years contributed to standardise for any such disparities. This is depicted in S6, S7, and S8 Tables. Of the morbidities, ADHD, schizophrenia, and eczema were no longer significant whilst iron deficiency anaemia was considered significant following adjustment for these factors. Of the cancers, only cervical cancer had a significantly reduced OR in individuals with DS following adjustment. The largest cause of cervical cancer is infection with human papillomavirus (HPV) [57]. Fitzpatrick et al. found that incidence of HPV infection, as well as other sexually transmitted infections (STIs), was significantly reduced in individuals with Down syndrome, which they posit could be due to “noted delays in sexual maturity” [58]. These adjusted findings suggest that smoking and/or ethnicity may be important mediators of disease burden of Down syndrome, especially cancers. This is in line with existing understanding of smoking as a major carcinogen [59].

### The study period

Individuals with DS, on average, contributed fewer years of follow-up compared with matched controls. Consequently, it is arguable that this differential loss to follow up may result in under-estimation of morbidity among individuals with DS. However, this difference was largely driven by increased rates of mortality among individuals with DS, compared to controls. Therefore, adjustments for loss to follow-up were not considered in primary analyses due to the impossibility of an intervention which prevents death. The present study used a fixed cohort (those alive at the start of follow-up) for simplicity and because the focus of this work is to inform public health service planning, not to draw inferences about hypothetical interventions. This analytic decision does pose a risk of survival bias, where healthier individuals are more likely to survive and contribute additional person-time. Conversely, it is also plausible that individuals with DS interact with health services more frequently than controls and are more likely to be diagnosed with a condition of interest due to those interactions, such as through active health surveillance.

Exploring the point prevalence of morbidities within discrete age bands over the course of follow-up was out of scope for the present study. The presentation of age-specific prevalence estimates should be a focus of future work, to account for potential nuances in prevalence which are obscured by a 20-year period prevalence.

### Generalisability of the findings & missingness in the dataset

The linked dataset is known to be broadly representative of the UK population in terms of age, sex, ethnicity, BMI and mortality. However, it is not established whether our DS cohort is broadly representative of individuals with DS in the UK or further afield. There are slight statistically significant differences between our DS population and the control group demographic comparisons, as depicted in Table 1. Matching is not guaranteed to create a perfectly balanced comparator population. These disparities introduce the possibility of residual confounding, such as in the incidence of age-related conditions such as dementia, potentially exaggerating or attenuating observed effects.

Within the linked dataset there is variation in the completeness of data across patients, with some variables being less complete than others. For example, in the HES dataset, information on age, sex and clinical characteristics are well reported but ethnicity is not [25]. In some cases, inconsistencies in data can impede accurate research [60,61]. However, linking multiple datasets, as done in this study, reduces the degree of missingness for some variables, for example combining CPRD and HES increases the completeness of the ethnicity variable from 78% to 97% [62].

### Comparison with other studies, DS-associated morbidity

In general, the findings of this study are in-line with existing publications which explore the prevalence of morbidities in individuals with DS, compared to matched controls. As expected, the “well-established” DS-associated morbidities, such as hypothyroidism, congenital heart disease, epilepsy, hearing impairment and dementia were significantly more common in the DS cohort versus matched controls, and the figures overlap with existing reports of population prevalence.

However, for a small number of morbidities our findings differ from existing studies. These discrepancies tended to occur for the morbidities where existing evidence is scant, and where a relationship between DS and the morbidity is not well established.

For example, the period prevalence of ADHD among children with DS in this study (1.5%), is much lower than reported by Oxelgren et al. (34.0%) [63] and Ekstein et al. (43.9%) [64]. This is also true for autism (6.3%), with existing reports of prevalence of up to 42% [65–68]. These significant differences are likely explained by differences in study design. Those existing studies, which report a much higher prevalence of autism and ADHD, actively screened for these conditions in DS cohorts. Screening for ADHD and autism is not routinely included in existing DS health surveillance guidelines [52], which may contribute to under-diagnosis in our study population. Several studies have suggested that behavioural disorders, such as autism and ADHD, are indeed underdiagnosed and undertreated among children with DS, due to a combination of atypical presentations and “diagnostic over-shadowing” [69,70]. Similarly, there is a risk that diagnoses of Down Syndrome Regression Disorder are masked by a diagnosis of dementia, falsely inflating the prevalence of dementia [71].

While some studies have reported a prevalence of sleep disordered breathing (SDB) among individuals with DS ranging between 43–63% [72–75], the period prevalence of SDB in this study was much lower (8.7% in adults & children combined, 19.1% among children only). Several studies have demonstrated that self or parental report of symptoms is insufficient to identify all cases of SDB [75,76] and again, SDB is not routinely included in existing DS health surveillance guidelines. Thus, the relatively lower prevalence of SDB in our study population could again be secondary to under-diagnosis, as opposed to true absence of disease.

In contrast, the period prevalence of inflammatory bowel disease (IBD) in the DS population in this study (8.0%) was higher than existing reports in the literature of around 2% [77]. This suggests that IBD may be more common in individuals with DS than previously thought, and it may in fact affect a relatively large number of patients.

This study also identified a high period prevalence of non-eczematous skin disorders (16.3%) in the DS cohort. This includes conditions such as psoriasis, lichen planus, pemphigoid, pemphigus, and vitiligo. It is noteworthy that these conditions, as well as IBD, are believed to have an underlying autoimmune aetiology [78,79]. Along with other immune mediated conditions, such as coeliac disease, type 1 diabetes mellitus, hyperthyroidism, and hypothyroidism, it appears that disorders of autoimmunity contribute a significant burden of disease in our DS cohort. This is in keeping with existing hypotheses regarding immune dysfunction in DS [80–83].

In both the primary and subgroup analysis, individuals with DS had a significantly increased period prevalence of non-accidental injury or maltreatment, compared with matched controls. While there are numerous studies suggesting that the prevalence of maltreatment is higher among children with intellectual disability [84–88], literature specific to DS is scant. There are two existing studies which show either no increased risk of maltreatment among children with DS, compared to the general population [89] or no difference after adjustment for confounding factors [90]. The absolute difference in the prevalence of maltreatment between our DS cohort and matched controls may be small but it is significant and adds to the limited existing evidence base on this topic. Coding and reporting practices may inflate differences; nevertheless, the findings warrant safeguarding awareness and better prospective data.

As described above, in the primary analysis, there were only two morbidities which appeared to occur *less* commonly in the DS cohort, compared with matched controls (anxiety/depression and ischaemic heart disease (IHD) in older individuals).

Several studies have already suggested lower rates of atherosclerosis, and coronary artery disease among individuals with DS [40,41]. In this study, the significant difference was only apparent when limiting the cohort to those aged over 40 years at start of follow-up. This likely reflects the fact that IHD tends to be a disease of older age.

It is also noteworthy that individuals with DS were significantly less likely than the control group to have a record of depression or anxiety. Our reported prevalence of depression and anxiety in the DS cohort is similar to that reported in the existing literature [91,92]. However, mood disorders may present differently in individuals with DS, compared to the general population. It is uncertain whether this, combined with difficulties in accessing healthcare or expressing symptoms, may have led to underdiagnoses of depression and anxiety in the DS cohort, as opposed to a truly reduced prevalence of mood disorders in this group. Lower recorded rates may reflect diagnostic overshadowing for mood disorders; alternatively, DS-specific physiological or lifestyle factors might be protective. Future work should aim to disentangle measurement from the condition.

Compared with the existing literature, the findings of this study are a significant contribution to the available evidence on the occurrence of multiple comorbidities in the DS population. Our findings add to the literature on the association, or lack thereof, between DS and ADHD, autism, chronic kidney disease, diabetes mellitus, Duchenne muscular dystrophy, inflammatory bowel disease, non-accidental injury, vitamin D deficiency, and schizophrenia.

### Comparison with other studies, Cancer

In general, the findings of this study complement existing publications describing the prevalence of cancers in individuals with DS. However, our findings add to the scant, and often contradictory, reports of “non-leukaemic cancers” among individuals with DS.

As described in the introduction, there is ongoing controversy about the prevalence and relative risk of solid tumours among individuals with DS. The findings of this study would support the existing literature that suggests an increased prevalence of testicular cancer among males with DS, compared to the general population [17,19]. This study, however, also supports the hypothesis that individuals with DS have a lower prevalence of several solid tumours (i.e., breast, cervical, colorectal, lung, melanoma, non-melanomatous skin, ovarian, prostate, uterine and Wilms’ tumour). A small number of studies have suggested increased risks of liver, gastric, ovarian cancer, retinoblastoma and lymphoma, among individuals with DS. However, the findings of this study did not support these associations.

A significantly reduced prevalence of non-melanomatous skin cancer (e.g., basal cell and squamous cell carcinomas) was observed in the DS cohort, compared to matched controls. To the best of our knowledge this association has not previously been described in the literature. However, this difference may be explained by other confounding factors for which it was not possible to adjust, such as time spent outdoors and sunlight exposure.

### Implications for practice and research

The use of a 20-year period prevalence offers valuable insights for current service planning in the healthcare of individuals with DS, particularly DS health surveillance. The morbidities which are core components of current DS health surveillance practice – congenital heart disease, leukaemia, thyroid dysfunction, and vision and hearing impairment [52–54] – were all common in our DS cohort, and significantly more common compared to matched controls, providing further evidence to support these practices.

Additionally, there were further morbidities which occurred commonly in our cohort and that could be amenable to screening and therapy. These include disorders of skin, arthritis, dementia, sleep disordered breathing, diabetes mellitus (combined), inflammatory bowel disease, chronic kidney disease, and ischaemic heart disease in older age groups, all occurring in greater than 5% of individuals with DS. Furthermore, the findings suggest an under-diagnosis of ADHD, autism and sleep disordered breathing, with a much lower period prevalence than would be expected based on existing estimates where active screening was employed.

These findings provide some evidence to support the expansion of health surveillance protocols for individuals with DS. This can include established diagnostic tests, such as HbA1c, fasting glucose, renal function tests, and polysomnography, targeted history taking, clinical examinations to identify signs and symptoms of inflammatory bowel disease, skin disease and arthritis, and cognitive or behavioural assessments to detect ADHD, autism, and early signs of dementia. The stratification of prevalence in the paediatric and adult populations is valuable in informing focused health surveillance in the paediatric DS population. Our findings add further evidence for surveillance of leukaemia in children with DS, such as blood testing in the first few days of life to screen for signs of Transient Leukaemia of Down Syndrome (TL-DS), as is done in the UK [93]. However, it must also be acknowledged that screening for disease is not based on population prevalence alone. Additional evidence is needed to satisfy the Wilson and Junger criteria [94], as well as informing when and how best to test for these conditions.

It is both noteworthy and concerning that the findings also suggest that children with DS are more likely to have a record of non-accidental injury or maltreatment, compared with matched controls. This is further evidence to support a high degree of vigilance among clinicians and other professionals caring for children with DS for the signs of child maltreatment.

Individuals with DS have access to standard national cancer screening programmes in the UK, which include surveillance for breast, colorectal and cervical cancer in adulthood. Some existing publications suggest that, for some cancers, individuals with DS may not require any, or at least less frequent, screening [95]. The findings of this study provide some support for amendments to the recommendations for cervical cancer screening among women with DS, to reflect their lower prevalence of disease. Conversely, our findings support the assertion that males with DS may benefit from *additional* screening for testicular cancer [17]. However, our findings do not provide support for the reduction or cessation of screening for breast and colorectal cancers among individuals with DS [17]. It should be noted that the findings of this study cannot be used in isolation to make decisions about cancer health surveillance in the individuals with DS, but they do contribute to the weight of available evidence.

### Unanswered questions and future research

Our research functions as a scoping project, identifying a difference in a 20-year period prevalence in multiple morbidities between individuals with and without DS. Whilst not within the scope of this study, age-specific prevalence estimates would provide a deeper insight into the development of certain morbidity differences at distinct stages of the lifespan. This would be a worthwhile avenue of future research to further guide age-appropriate DS health surveillance.

Our analysis of childhood prevalence provided an illustration of early-onset morbidities and cancers in the paediatric DS population. However, it was not within the remit of this study to examine changes in the prevalence or incidence of DS-associated morbidities or cancers and the distribution in the age of onset across the entire DS life course. Such evidence would be valuable to inform health surveillance guidelines, as it may be that screening for some conditions is only required during certain periods of life. This could reduce unnecessary screening for some and increase the efficiency of resource allocation.

Furthermore, it was not within the remit of this study to determine which of the morbidities or cancers were diagnosed via health surveillance, and which were picked up through other means (e.g., symptomatic presentation). Such information could inform the efficacy of current health surveillance practices and inform their improvement. Such research would require a different dataset or methodological approach, such as a prospective observational study.

While we propose that the findings of this study support the expansion of DS health surveillance guidelines to include a wider range of morbidities it is not within the remit of this study to assess the acceptability, or reliability, of specific approaches to screening. Some health surveillance practices (e.g., overnight polysomnography for the diagnosis of sleep disordered breathing) can be expensive, difficult to access and poorly tolerated. Future studies could explore the realities of delivering health surveillance protocols on the front line, in terms of access and acceptability.

Furthermore, while our findings may support a change to some aspects of routine cancer surveillance among individuals with DS, our findings cannot be used in isolation to revise guidelines. Future studies could explore the sensitivity and specificity of cancer screening, particularly solid tumours, in individuals with DS and assess the impact on mortality and morbidity.

Our study also found a significantly increased mortality within the study period, in the DS cohort compared with matched controls. It was not within the remit of study to examine the causes of death in either group, but this data can be accessed within the larger linked dataset. Future research could explore the age and causes of death in this substantial DS cohort and compare with existing literature. It would also be valuable to explore what contribution multi-morbidity has to mortality among individuals with DS.

Finally, while this study describes the period prevalence of morbidities among a large cohort of individuals with DS, it cannot capture the impact of these conditions on overall health and wellbeing. It may be that some morbidities, while less prevalent than others in the DS cohort, do in fact have a significant impact on the quality of life of patients and carers. Future research could employ a qualitative approach to explore the realities of living with these morbidities, differences in treatment outcomes compared to individuals without DS, and the health priorities of patients and carers.

## Supporting information

S1 Appendix Methodology. Datasets. (DOCX)

S1 File References. (DOCX)

S1 Table Coding list for Down syndrome. (DOCX)

S2 Table The DS-associated morbidities and cancers investigated in the dataset. (DOCX)

S3 Table Primary analysis (adults and children). Summarising and comparing the study period prevalence and odds ratios (OR) of DS-associated morbidities in the DS cohort v. the matched control group.
(DOCX)

S4 Table Primary analysis (adults & children): Summarising and comparing the study period prevalence and odds ratios (OR) of cancers in the DS cohort v. the matched control group. (DOCX)

S5 Table Subgroup analysis (children only): Summarising and comparing the period prevalence and odds ratios (OR) of DS-associated morbidities and cancers in the DS cohort v. the matched control group. (DOCX)

S6 Table Primary analysis (adults & children): Further adjusted odds ratios (aOR) for the occurrence of DS-associated morbidities in the DS cohort v. matched controls. (DOCX)

S7 Table Primary analysis (adults & children): Further adjusted odds ratios (aOR) for the occurrence of cancers in the DS cohort v. matched controls. (DOCX)

S8 Table Subgroup analysis (children only): Further adjusted odds ratios (aOR) for the occurrence of DS-associated morbidities and cancers in the DS cohort v. matched controls. (DOCX)
