# Supplementary material for: Prevalence of multiple morbidities and cancers in individuals with Down syndrome: A matched descriptive study using linked electronic health record data
Source: PLoS One. 2026 Jun 3;21(6):e0349794. doi: 10.1371/journal.pone.0349794 (PMC13232805; doi:10.1371/journal.pone.0349794)
Supplement: S1 Appendix — (DOCX) [file pone.0349794.s001.docx]

**S1 Appendix - Methodology**

**Secondary care data: Hospital Episode Statistics (HES)**

HES provides information on hospital admissions, outpatient appointments, procedures, and A&E attendances for eligible patients in England. HES covers all NHS trusts in England, including mental health Trusts. Data is extracted for the purpose of hospital reimbursement under Payment by Results (PbR).

Diagnoses are coded using the hierarchical coding scheme, International Statistical Classification of Diseases and Health-Related Problems, 10th revision (ICD-10). The ICD-10 coding system includes codes for disease, signs, symptoms, abnormal findings, social circumstances and external causes of injury or disease.

A discharge summary produced upon discharge from hospital is forwarded to the clinical coding department who input the information on the local patient information database. Clinical coders undergo an accredited training programme and follow standardised rules for translating information into clinical codes [25, 96, 97]. Discharge letters, clinical letters and referral letters are ideally copied to the primary care physician and transcribed onto the primary care (CPRD) dataset, as described above. However, in practice, this is not always the case and the linkage of HES and CPRD therefore provides a more complete picture of patient journey. The HES dataset has been used extensively used for epidemiological research [98-100] and validated for several conditions [101-103].

**National Cancer Registration and Analysis Service (NCRAS)**

Data from primary care (CPRD) and secondary care (HES) is linked to the NCRAS, provided by Public Health England (PHE), via the NCRAS. This dataset includes a record of all registerable tumours diagnosed or treated in England, of which NCRAS has been notified [104].

Cancers are coded using the International Classification of Diseases for Oncology, revision 3, 2011 [105] and ‘back mapped’ to the tenth revision of the International Classification of Diseases version 10 (ICD-10). Registrable conditions are broadly: all invasive tumours, all uncertain behaviour tumours, all in situ tumours and benign tumours within the brain or central nervous system [26].

Estimates of data completeness are high (estimate >99%), as the registry is population based and receives death certificates [26].

**Office of National Statistics (ONS)**

ONS data, which is also linked to CPRD, provides information on the causes of mortality of all patients in England, and the official date of death. Cause-specific mortality data is extracted from death certifications and coded using ICD-10.

The ONS also provides the IMD and Townsend Score [106, 107], based on the patient and/or practice postcode. This information is poorly recorded elsewhere in the linked dataset and provides a measure of socio-economic status [104].

In CALIBER, the creation of code lists is assisted by access to the CALIBER Portal, an online resource for clinical research using electronic health records. The portal provides access to code lists which have been compiled and utilised by other researchers undertaking research on the CPRD and HES datasets. When a CALIBER researcher is examining a condition of interest, not currently included on the Data Portal, their code is made available for use by future researchers, following peer review [108].
