# Supplementary material for: Prevalence of multiple morbidities and cancers in individuals with Down syndrome: A matched descriptive study using linked electronic health record data
Source: PLoS One. 2026 Jun 3;21(6):e0349794. doi: 10.1371/journal.pone.0349794 (PMC13232805; doi:10.1371/journal.pone.0349794)
Supplement: S1 File — (DOCX) [file pone.0349794.s002.docx]

1. NHS Digital. Hospital Episode Statistics. 2017. Available from: <http://content.digital.nhs.uk/hes2017>
2. Health and Social Care Information Centre (HSCIC). National clinical coding standards ICD-10. 2017. <https://isd.hscic.gov.uk/trud3/user/guest/group/0/pack/28>
3. Jeevan R, Cromwell D, Trivella M, Lawrence G, Kearins O, Pereira J, et al. Reoperation rates after breast conserving surgery for breast cancer among women in England: Retrospective study of hospital episode statistics. *BMJ.* 2012;345:e4505. doi:10.1136/bmj.e4505
4. Patel H, Bell D, Molokhia M, Srishanmuganathan J, Patel M, Car J, et al. Trends in hospital admissions for adverse drug reactions in England: analysis of national hospital episode statistics 1998-2005. *BMC Clin Pharmacol.* 2007;7:9. doi:10.1186/1472-6904-7-9
5. Aylin P, Alexandrescu R, Jen M, Mayer E, Bottle A. Day of week of procedure and 30 day mortality for elective surgery: retrospective analysis of hospital episode statistics. *BMJ.* 2013;346:f2424. doi:10.1136/bmj.f2424
6. Thorn JC, Turner E, Hounsome L, Walsh E, Donovan JL, Verne J, et al. Validation of the Hospital Episode Statistics Outpatient Dataset in England. *PharmacoEconomics.* 2016;34(2):161–8. doi:10.1007/s40273-015-0326-3
7. Britton A, Milne B, Butler T, Sanchez Galvez A, Shipley M, Rudd A, et al. Validating self reported strokes in a longitudinal UK cohort study (Whitehall II): Extracting information from hospital medical records versus the Hospital Episode Statistics database. *BMC Med Res Methodol.* 2012;12:83. doi:10.1186/1471-2288-12-83
8. Tovikkai C, Charman SC, Praseedom RK, Gimson AE, Watson CJ, Copley LP, van der Meulen J. Linkage of a national clinical liver transplant database with administrative hospital data: Methods and validation. *Transplantation.* 2014;98(3):341–7. doi:10.1097/TP.0000000000000128
9. Clinical Practice Research Datalink (CPRD). CPRD linked data. 2017. <http://www.cprd.com/dataAccess/linkeddata.asp>
10. World Health Organization. International classification of diseases for oncology (ICD O) (3rd ed., 1st rev.). Geneva: World Health Organization; 2013. Available from: <https://iris.who.int/handle/10665/96612>
11. McLennan D, Barnes H, Noble M, Davies J, Garratt E, Dibben C. The English Indices of Deprivation 2010. Crown; 2011. Available from: <https://www.gov.uk/government/uploads/system/uploads/attachment_data/file/6320/1870718.pdf>
12. Department for Communities and Local Government. The English Indices of Deprivation 2007: Technical report. London: Department for Communities and Local Government; 2007. <https://gisinfo.hertfordshire.gov.uk/GISdata/iod/IoD2007_TechReport.pdf>
13. Denaxas S, Gonzalez-Izquierdo A, Direk K, Fitzpatrick NK, Fatemifar G, Banerjee A, et al. UK phenomics platform for developing and validating electronic health record phenotypes: CALIBER. *J Am Med Inform Assoc.* 2019;26(12):1545–59. doi:10.1093/jamia/ocz105
14. Ioannidis JPA. Why most published research findings are false. *PLoS Med.* 2005;2(8):e124.
15. Aslam AA, Baksh RA, Pape SE, Strydom A, Gulliford MC, Chan LF; GO-DS21 Consortium. Diabetes and obesity in Down syndrome across the lifespan: a retrospective cohort study using U.K. electronic health records. *Diabetes Care.* 2022;45(12):2892-2899. doi:10.2337/dc22-0482.
16. NHS England. Commissioning medicines for children in specialised services. London: NHS England; 2024 Mar. Available from: <https://www.england.nhs.uk/wp-content/uploads/2017/03/commissioning-medicines-for-children-in-specialised-services-v0.3.pdf>
17. General Medical Council. *Definitions of children, young people and parents*. London: GMC; 2024. Available from: <https://www.gmc-uk.org/professional-standards/the-professional-standards/protecting-children-and-young-people/definitions-of-children-young-people-and-parents>
18. Children Act 1989. London: HMSO; 1989. c.41.
