# Supplementary material for: Prevalence of multiple morbidities and cancers in individuals with Down syndrome: A matched descriptive study using linked electronic health record data
Source: PLoS One. 2026 Jun 3;21(6):e0349794. doi: 10.1371/journal.pone.0349794 (PMC13232805; doi:10.1371/journal.pone.0349794)
Supplement: S1 Table — (DOCX) [file pone.0349794.s003.docx]

**S1 Table – Coding List for Down syndrome**

| **Down’s syndrome READ Codes in the CPRD GOLD Database** | **Down’s syndrome ICD-10 Codes**  **in the HES APC database:** |
| --- | --- |
| PJ0..00 Down's syndrome - trisomy 21 | Q90 Down syndrome |
| PJ0..11 Mongolism | Q90.0 Trisomy 21, meiotic nondisjunction |
| PJ0..12 Trisomy 21 | Q90.1 Trisomy 21, mosaicism (mitotic nondisjunction) |
| PJ00.00 Trisomy 21, meiotic nondisjunction | Q90.2 Trisomy 21, translocation |
| PJ01.00 Trisomy 21, mosaicism | Q90.9 Down syndrome, unspecified |
| PJ01.11 Trisomy 21, mitotic nondisjunction |  |
| PJ02.00 Trisomy 21, translocation |  |
| PJ02.11 Partial trisomy 21 in Down's syndrome |  |
| PJ0z.00 Down's syndrome NOS |  |
| PJ0z.11 Trisomy 21 NOS |  |
