# Supplementary material for: Prevalence of multiple morbidities and cancers in individuals with Down syndrome: A matched descriptive study using linked electronic health record data
Source: PLoS One. 2026 Jun 3;21(6):e0349794. doi: 10.1371/journal.pone.0349794 (PMC13232805; doi:10.1371/journal.pone.0349794)
Supplement: S2 Table — (DOCX) [file pone.0349794.s004.docx]

**S2 Table: The DS-associated morbidities and cancers investigated in the dataset**

| **Cardiovascular** | Congenital cardiac disease  Ischaemic heart disease  Stroke |
| --- | --- |
| **Ear, Nose & Throat** | Hearing impairment  Sleep disordered breathing |
| **Vision / Ophthalmic** | Glaucoma |
| **Neurological** | Epilepsy |
| **Psychiatric/ Behaviour** | ADHD  Anxiety & depression  Autism  Dementia  Schizophrenia |
| **Gastrointestinal / Renal** | Chronic kidney disease  Congenital gastrointestinal disease  Gastro-oesophageal reflux  Inflammatory bowel disease |
| **Endocrine / Autoimmune** | Coeliac disease  Hyperthyroidism  Hypothyroidism  Type 1 diabetes mellitus  Type 2 diabetes mellitus  Diabetes mellitus (combined) |
| **Haematological** | Iron deficiency |
| **Musculoskeletal** | Arthritis (combined)  Duchene muscular dystrophy |
| **Dermatological** | Eczema  Skin disorders, non-eczema (combined) |
| **Other** | Non-accidental injury / maltreatment  Undescended testis  Vitamin D deficiency |
| **Cancers** | Bladder  Bone  Brain/ CNS  Breast  Cervix  Colorectal  Gastro-oesophageal  Leukaemia  Liver / hepatobiliary  Lung  Lymphoma  Melanoma  Myeloma  Neuroblastoma  Ovarian  Pancreas  Prostate  Renal  Retinoblastoma  Skin, non-melanomatous  Testicular  Thyroid & parathyroid  Uterus  Wilms’ |

Nb. Each health condition of interest is defined using a phenotyping code list. This process is described in the Methods. The list of codes used to define each condition is included in S1 Table.
