# Supplementary material for: Prevalence of multiple morbidities and cancers in individuals with Down syndrome: A matched descriptive study using linked electronic health record data
Source: PLoS One. 2026 Jun 3;21(6):e0349794. doi: 10.1371/journal.pone.0349794 (PMC13232805; doi:10.1371/journal.pone.0349794)
Supplement: S3 Table — Summarising and comparing the study period prevalence and odds ratios (OR) of DS-associated morbidities in the DS cohort v. the matched control group. (DOCX) [file pone.0349794.s005.docx]

S3 Table: Primary analysis (adults & children): Summarising and comparing the study period prevalence and odds ratios (OR) of DS-associated morbidities in the DS cohort v. the matched control group.

| **Morbidity** | **DS Cohort**  **N=** **4,648** | | **Matched Control Group**  **N=** **23,238** | | **p-value***  **(p<0.01)** | **OR (CI)**  **(95% CI >1)**  *(95% CI <1)* |
| --- | --- | --- | --- | --- | --- | --- |
|  | **n** | **% (95% CI)** | **n** | **% (95% CI)** |  |  |
| ADHD | 59 | 1.3% (1.0%-1.6%) | 159 | 0.7% (0.6%-0.8%) | **<0.001** | **1.9 (1.4 -2.5)** |
| Anxiety/depression | 657 | 14.1% (13.2%-15.2%) | 4,969 | 21.4% (20.9%-21.9%) | **<0.001** | *0.6 (0.6-0.7)* |
| Arthritis (combined) | 570 | 12.3% (11.4%-13.2%) | 2,906 | 12.5% (12.1%-12.9%) | 0.648 | 1.0 (0.9-1.1) |
| Atlantoaxial instability | 39 | 0.8% (0.6%-1.2%) | 26 | 0.1% (0.1%-0.2%) | **<0.001** | **7.6 (4.6-12.4)** |
| Autism | 230 | 5.0% (4.4%-5.6%) | 160 | 0.7% (0.6%-0.8%) | **<0.001** | **7.5 (6.4-8.0)** |
| Chronic kidney disease | 300 | 6.5% (5.8%-7.2%) | 645 | 2.8% (2.6%-3.0%) | **<0.001** | **2.4 (2.1-2.8)** |
| Coeliac disease | 100 | 2.2% (1.8%-2.6%) | 87 | 0.4% (0.3%-0.5%) | **<0.001** | **5.9 (4.4-7.8)** |
| Congenital cardiac disease | 1,293 | 27.8% (26.6%-29.1%) | 207 | 0.9% (0.8%-1.0%) | **<0.001** | **42.9 (36.9-49.9)** |
| Congenital gastrointestinal disease | 106 | 2.3% (1.9%-2.8%) | 57 | 0.3% (0.2%-0.3%) | **<0.001** | **9.5 (6.9-13.1)** |
| Dementia | 816 | 17.6% (16.5%-18.7%) | 168 | 0.7% (0.6%-0.8%) | **<0.001** | **29.2 (24.7-34.6)** |
| Dementia (≥30yrs at start of follow-up)” | 807 | 37.3% (35.3%-39.4%) | 158 | 1.5% (1.3%-1.8%) | **<0.001** | **38.4 (32.1-45.9)** |
| Diabetes Mellitus (combined) | 375 | 8.1% (7.3%-8.9%) | 1,394 | 6.0% (5.7%-6.3%) | **<0.001** | **1.4 (1.2-1.6)** |
| Diabetes Mellitus, Type 1^ | 56 | 1.2% (0.9%-1.6%) | 101 | 0.4% (0.4%-0.5%) | **<0.001** | **2.8 (2.0-3.9)** |
| Diabetes Mellitus, Type 2^ | 145 | 3.1% (2.7%-3.7%) | 784 | 3.4% (3.2%-3.6%) | 0.378 | 0.9 (0.8-1.1) |
| Duchenne muscular dystrophy | 4 | 0.1% (0.0%-0.2%) | 18 | 0.1% (0.1%-0.1%) | 0.849‡ | 1.1 (0.4-3.3) |
| Eczema | 1,354 | 29.1% (27.8%-30.5%) | 5,504 | 23.7% (23.1%-24.2%) | **<0.001** | **1.3 (1.2-1.4)** |
| Epilepsy | 1,018 | 21.9% (20.7%-23.1%) | 598 | 2.6% (2.4%-2.8%) | **<0.001** | **10.6 (9.5-11.8)** |
| Gastro-oesophageal reflux | 576 | 12.4% (11.5%-13.4%) | 2,154 | 9.3% (8.9%-9.7%) | **<0.001** | **1.4 (1.3-1.5)** |
| Glaucoma | 38 | 0.8% (0.6%-1.1%) | 254 | 1.1% (1.0%-1.2%) | 0.092 | 0.8 (0.5-1.1) |
| Hearing impairment | 890 | 19.2% (18.0%-20.3%) | 856 | 3.7% (3.5%-3.9%) | **<0.001** | **6.2 (5.6-6.8)** |
| Hyperthyroidism | 150 | 3.2% (2.8%-3.8%) | 214 | 0.9% (0.8%-1.1%) | **<0.001** | **3.6 (2.9-4.4)** |
| Hypothyroidism | 1,413 | 30.4% (29.1%-31.7%) | 738 | 3.2% (3.0%-3.4%) | **<0.001** | **13.3 (12.1-14.7)** |
| Inflammatory bowel disease | 371 | 8.0% (7.2%-8.8%) | 772 | 3.3% (3.1%-3.6%) | **<0.001** | **2.5 (2.2-2.8)** |
| Iron deficiency anaemia | 215 | 4.6% (4.1%-5.3%) | 907 | 3.9% (3.7%-4.2%) | 0.022 | 1.2 (1.0-1.4) |
| Ischaemic heart disease (IHD) | 269 | 5.8% (5.2%-6.5%) | 1,134 | 4.9% (4.6%-5.2%) | 0.010 | 1.2 (1.0-1.4) |
| IHD (≥40yrs at start of follow-up)’ | 144 | 9.6% (8.2%-11.2%) | 911 | 13.8% (13.0%-14.7%) | **<0.001** | *0.7 (0.6-0.8)* |
| Non-accidental injury/ maltreatment | 108 | 2.3% (1.9%-2.8%) | 284 | 1.2% (1.1%-1.4%) | **<0.001** | **1.9 (1.5-2.4)** |
| Schizophrenia | 39 | 0.8% (0.6%-1.2%) | 124 | 0.5% (0.5%-0.6%) | 0.013 | **1.6 (1.1-2.3)** |
| Skin disorders, non-eczema | 756 | 16.3% (15.2%-17.4%) | 1,742 | 7.5% (7.2%-7.8%) | <0.001 | **2.4 (2.2-2.6)** |
| Sleep disordered breathing | 402 | 8.7% (7.9%-9.5%) | 537 | 2.3% (2.1%-2.5%) | **<0.001** | **4.0 (3.5-4.6)** |
| Stroke | 173 | 3.7% (3.2%-4.3%) | 443 | 1.9% (1.7%-2.1%) | **<0.001** | **2.0 (1.7-2.4)** |
| Undescended testis | 146 | 3.1% (2.7%-3.7%) | 169 | 0.7% (0.6%-0.9%) | **<0.001** | **4.4 (3.5-5.5)** |
| Vitamin D deficiency | 73 | 1.6% (1.3%-2.0%) | 181 | 0.8% (0.7%-0.9%) | **<0.001** | **2.0 (1.6-2.7)** |
|  |  |  |  |  |  |  |

Nb. Cases (individuals with DS) are matched with at least 4 matched controls (non-DS individuals) based on GP practice, practice level index of multiple deprivation, year of birth ± 1 year, sex, and index date.

ADHD: Attention Deficit Hyperactivity Disorder, IHD: Ischaemic heart disease, OR = odds ratio; CI = 95% confidence intervals

*p values calculated using χ2 (comparison of proportions)

‡p value calculated using Fisher’s exact test (comparison of proportions, non-parametric)

^The prevalence of type 1 and type 2 diabetes (separately) is based on CPRD data only.

“DS N=2,163, Controls N=10,346

‘DS N=1,501, Controls N=6,596

*Start of follow-up is defined as the latest of the patient registration date, the practice UTS date, and 01/01/1998*
