# Supplementary material for: Prevalence of multiple morbidities and cancers in individuals with Down syndrome: A matched descriptive study using linked electronic health record data
Source: PLoS One. 2026 Jun 3;21(6):e0349794. doi: 10.1371/journal.pone.0349794 (PMC13232805; doi:10.1371/journal.pone.0349794)
Supplement: S4 Table — (DOCX) [file pone.0349794.s006.docx]

**S4 Table: Primary analysis (adults & children): Summarising and comparing the study period prevalence and odds ratios (OR) of cancers in the DS cohort v. the matched control group.**

| **Cancer site** | **DS Cohort**  **(N=4,648)** | **Matched Control Group**  **(N=23,238)** | | | | **p-value***  **(p<0.01)** | **OR (CI)**  **(95% CI >1)**  *(95% CI <1)* |
| --- | --- | --- | --- | --- | --- | --- | --- |
|  | **n** | **%(95% CI)** | **n** | **%(95% CI)** |  | |  |
| Bladder | 9 | 0.2% (0.1%-0.4%) | 64 | 0.3% (0.2%-0.4%) | 0.319 | | 0.7 (0.4-1.4) |
| Bone | 4 | 0.1% (0.0%-0.2%) | 18 | 0.1% (0.1%-0.1%) | 0.849‡ | | 1.1 (0.4-3.3) |
| Brain/Central Nervous System | 15 | 0.3% (0.2%-0.5%) | 79 | 0.3% (0.3%-0.4%) | 0.853 | | 1.0 (0.6-1.7) |
| Breast | 16 | 0.3% (0.2%-0.6%) | 311 | 1.3% (1.2%-1.5%) | **<0.001** | | *0.3 (0.2-0.4)* |
| Cervix | 5 | 0.1% (0.0%-0.3%) | 429 | 1.9% (1.7%-2.0%) | **<0.001‡** | | *0.1 (0.1-0.2)* |
| Colorectal | 24 | 0.5% (0.4%-0.8%) | 413 | 1.8% (1.6%-2.0%) | **<0.001** | | *0.3 (0.2-0.4)* |
| Gastro-oesophageal | 6 | 0.1% (0.1%-0.3%) | 46 | 0.2% (0.2%-0.3%) | 0.321 | | 0.7 (0.3-1.5) |
| Leukaemia | 45 | 1.0% (0.7%-1.3%) | 38 | 0.2% (0.1%-0.2%) | **<0.001** | | **6.0 (3.9-9.2)** |
| Liver/biliary | 3 | 0.1% (0.0%-0.2%) | 15 | 0.1% (0.0%-0.1%) | 1.00‡ | | 1.0 (0.3-3.5) |
| Lung | 5 | 0.1% (0.0%-0.3%) | 116 | 0.5% (0.4%-0.6%) | **<0.001** | | *0.2 (0.1-0.5)* |
| Lymphoma | 12 | 0.3% (0.2%-0.5%) | 72 | 0.3% (0.3%-0.4%) | 0.557 | | 0.8 (0.5-1.5) |
| Melanoma | 6 | 0.1% (0.1%-0.3%) | 104 | 0.5% (0.4%-0.5%) | 0.002 | | *0.3 (0.1-0.7)* |
| Skin, non-melanoma | 27 | 0.6% (0.4%-0.9%) | 539 | 2.3% (2.1%-2.5%) | **<0.001** | | *0.3 (0.2-0.4)* |
| Myeloma | 0 | 0.0% (0.0%-0.0%) | 3 | 0.01% (0.0%-0.04%) | 0.439‡ | | - |
| Neuroblastoma | 0 | 0.0% (0.0%-0.0%) | 3 | 0.01% (0.0%-0.04%) | 0.439‡ | | - |
| Ovarian | 6 | 0.1% (0.1%-0.3%) | 84 | 0.34% (0.3%-0.5%) | 0.011 | | *0.4 (0.2-0.8)* |
| Pancreas | 6 | 0.1% (0.1%-0.3%) | 24 | 0.1% (0.1%-0.2%) | 0.624 | | 1.3 (0.5-3.1) |
| Prostate | 3 | 0.1% (0.0%-0.2%) | 132 | 0.6% (0.5%-0.7%) | **<0.001‡** | | *0.1 (0.0-0.4)* |
| Renal | 3 | 0.1% (0.0%-0.2%) | 33 | 0.1% (0.1%-0.2%) | 0.179‡ | | 0.5 (0.1-1.5) |
| Retinoblastoma | 0 | 0.0% (0.0%-0.0%) | 1 | 0.0% (0.0%-0.03%) | 0.655‡ | | - |
| Testicular | 20 | 0.4% (0.3%-0.7%) | 20 | 0.1% (0.1%-0.7%) | **<0.001** | | **5.0 (2.7-9.3)** |
| Thyroid/parathyroid | 5 | 0.1% (0.0%-0.3%) | 42 | 0.2% (0.1%-0.2%) | 0.267‡ | | 0.6 (0.2-1.5) |
| Uterus | 16 | 0.3% (0.2%-0.6%) | 308 | 1.3% (1.2%-1.5%) | **<0.001** | | *0.3 (0.2-0.4)* |
| Wilms’ tumour | 2 | 0.04% (0.0%-0.2%) | 30 | 0.1% (0.1%-0.2%) | 0.114‡ | | 0.3 (0.1-1.4) |
| Any of the cancers above | 217 | 4.7% (4.1%-5.3%) | 2,421 | 10.4% (10.0%-10.8%) | **<0.001** | | *0.4 (0.4-0.5)* |

Nb. Cases (individuals with DS) are matched with at least 4 matched controls (non-DS individuals) based on GP practice, practice level index of multiple deprivation, year of birth ± 1 year, sex, and index date.

*p Values calculated using χ2 (comparison of proportions). ‡p value calculated Fisher’s exact test (comparison of proportions, non-parametric)

- = unable to calculate odd ratios due to absence of cancer in cases and/or control.
