# Supplementary material for: Prevalence of multiple morbidities and cancers in individuals with Down syndrome: A matched descriptive study using linked electronic health record data
Source: PLoS One. 2026 Jun 3;21(6):e0349794. doi: 10.1371/journal.pone.0349794 (PMC13232805; doi:10.1371/journal.pone.0349794)
Supplement: S5 Table — (DOCX) [file pone.0349794.s007.docx]

**S5 Table: Subgroup-analysis (children only): Summarising and comparing the period prevalence and odds ratios (OR) of DS-associated morbidities and cancers in the DS cohort v. the matched control group.**

| **Morbidity** | **DS Cohort, Children Only**  **N=1,340**  *n* (%)/average (95% CI) | | **Matched Control Group, Children Only**  **N=6,711**  *n* (%)/average (95% CI) | | **p-value***  **(p<0.01)** | **OR (CI)**  **(95% CI >1)**  *(95% CI <1)* |
| --- | --- | --- | --- | --- | --- | --- |
|  | **n** | **% (95% CI)** | **n** | **% (95% CI)** |  |  |
| ADHD | 20 | 1.5% (1.0%-2.3%) | 78 | 1.2% (0.9%-1.5%) | 0.314 | 1.3 (0.8-2.1) |
| Anxiety/depression | 25 | 1.9% (1.3%-2.8%) | 189 | 2.8% (2.5%-3.2%) | 0.048 | 0.7 (0.4-1.0) |
| Arthritis (combined) | 12 | 0.9% (0.5%-1.6%) | 26 | 0.4% (0.3%-0.6%) | 0.013 | **2.3 (1.2-4.6)** |
| Atlantoaxial instability | 11 | 0.8% (0.5%-1.5%) | 0 | 0.0% (0.0%-0.0%) | **<0.001‡** | - |
| Autism | 85 | 6.3% (5.2%-7.8%) | 89 | 1.3% (1.1%-1.6%) | **<0.001** | **5.0 (3.7-6.8)** |
| Chronic kidney disease | 15 | 1.1% (0.7%-1.9%) | 19 | 0.2% (0.2%-0.4%) | **<0.001** | **4.0 (2.0-7.9)** |
| Coeliac disease | 38 | 2.8% (2.1%-3.9%) | 17 | 0.3% (0.2%-0.4%) | **<0.001** | **11.5 (6.5-20.4)** |
| Congenital cardiac disease | 754 | 56.3% (53.6%-58.9%) | 72 | 1.1% (0.9%-1.4%) | **<0.001** | **118.6 (91.8-153.3)** |
| Congenital gastrointestinal disease | 63 | 4.7% (3.7%-6.0%) | 24 | 0.4% (0.2%-0.5%) | **<0.001** | **13.8 (8.6-22.1)** |
| Diabetes Mellitus (combined) | 37 | 2.8% (2.0%-3.8%) | 46 | 0.7% (0.5%-0.9%) | **<0.001** | **4.1 (2.7-6.4)** |
| Diabetes Mellitus, Type 1^ | 22 | 0.9% (0.4%-1.3%) | 9 | 0.7% (0.1%-0.3%) | 0.002 | **3.5 (1.5-8.2)** |
| Duchenne muscular dystrophy | 4 | 0.3% (0.1%-0.8%) | 3 | 0.04% (0.01%-0.14%) | 0.004‡ | **6.7 (1.5-30.0)** |
| Eczema | 323 | 24.1% (21.9%-26.5%) | 2119 | 31.6% (30.5%-32.7%) | **<0.001** | *0.7 (0.6-0.8)* |
| Epilepsy | 80 | 6.0% (4.8%-7.3%) | 110 | 1.6% (1.4%-2.0%) | **<0.001** | **3.8 (2.8-5.1)** |
| Gastro-oesophageal reflux | 254 | 19.0% (16.9%-21.%) | 280 | 4.2% (3.7%-4.7%) | **<0.001** | **5.4 (4.5-6.4)** |
| Glaucoma | 6 | 0.5% (0.2%-1.0%) | 3 | 0.04% (0.01%-0.14%) | **<0.001‡** | **10.1 (2.5-40.3)** |
| Hearing impairment | 315 | 23.5% (21.3%-25.9%) | 165 | 2.5% (2.1%-2.9%) | **<0.001** | **12.2 (10.0-14.9)** |
| Hyperthyroidism | 34 | 2.5% (1.8%-3.5%) | 1 | 0.01% (0.00%-0.11%) | **<0.001‡** | - |
| Hypothyroidism | 212 | 15.8% (14.0%-17.8%) | 32 | 0.5% (0.3%-0.7%) | **<0.001** | **39.2 (26.9-57.2)** |
| Inflammatory bowel disease | 113 | 8.4% (7.1%-10.1%) | 172 | 2.6% (2.2%-3.0%) | **<0.001** | **3.5 (2.7-4.5)** |
| Iron deficiency anaemia | 34 | 2.5% (1.8%-3.5%) | 84 | 1.3% (1.0%-1.6%) | **<0.001** | **2.1 (1.4-3.1)** |
| NAI/Maltreatment | 45 | 3.4% (2.5%-4.5%) | 139 | 2.1% (1.8%-2.4%) | 0.004 | **1.6 (1.2-2.3)** |
| Schizophrenia | 1 | 0.1% (0.01%-0.5%) | 5 | 0.1% (0.0%-0.2%) | 0.999‡ | 1.0 (0.1-8.6) |
| Skin disorders, non-eczema | 60 | 4.5% (3.5%-5.7%) | 171 | 2.6% (2.2%-3.0%) | **<0.001** | **1.8 (1.3-2.4)** |
| Sleep disordered breathing | 256 | 19.1% (17.1%-21.3%) | 104 | 1.6% (1.3%-1.9%) | **<0.001** | **15.0 (11.8-19.0)** |
| Stroke | 14 | 1.0% (0.6%-1.8%) | 5 | 0.1% (0.0%-0.2%) | **<0.001‡** | **14.2 (5.1-39.4)** |
| Undescended testis | 72 | 5.4% (4.3%-6.7%) | 102 | 1.5% (1.3%-1.8%) | **<0.001** | **3.7 (2.7-5.0)** |
| Vitamin D deficiency | 20 | 1.5% (1.0%-2.3%) | 78 | 0.6% (0.5%-0.8%) | **0.001** | **2.5 (1.4-4.2)** |
| **Cancers** |  |  |  |  |  |  |
| Leukaemia | 29 | 2.2% (1.5%-3.1%) | 2 | 0.03% (0.01%-0.12%) | **<0.001‡** | 74.2 (17.7-311.4) |
| Lymphoma | 3 | 0.2% (0.1%-0.7%) | 7 | 0.1% (0.1%-0.2%) | 0.257‡ | 2.2 (0.6-8.3) |
| Neuroblastoma | 0 | 0.0% (0.0%-0.0%) | 2 | 0.03% (0.01%-0.12%) | 0.527‡ | - |

Nb. Cases (individuals with DS) are matched with at least 4 matched controls (non-DS individuals) based on GP practice, practice level index of multiple deprivation, year of birth ± 1 year, sex, and index date.

Children were defined as those individuals who were aged ≤18 years at the end of follow-up.

End of follow-up is defined as the earliest of the patient transfer out date, the practice last collection date, date of death and 31/12/2017.

ADHD: Attention Deficit Hyperactivity Disorder, CI = 95% confidence intervals, NAI: Non-accidental injury, OR = odds ratio

*p values calculated using χ2 (comparison of proportions).

‡p value calculated Fisher’s exact test (comparison of proportions, non-parametric)

^The prevalence of type 1 and type 2 diabetes (separately) is based on CPRD data only.
