# Supplementary material for: Prevalence of multiple morbidities and cancers in individuals with Down syndrome: A matched descriptive study using linked electronic health record data
Source: PLoS One. 2026 Jun 3;21(6):e0349794. doi: 10.1371/journal.pone.0349794 (PMC13232805; doi:10.1371/journal.pone.0349794)
Supplement: S6 Table — (DOCX) [file pone.0349794.s008.docx]

S6 Table: Primary analysis (adults & children): Further adjusted odds ratios (aOR) for the occurrence of DS-associated morbidities in the DS cohort v. matched controls.

| **Morbidity** | **aOR (CI)**  **(95% CI </>1)** |
| --- | --- |
|  |  |
| ADHD | 1.41(0.86-2.31) |
| Anxiety/depression | **0.68(0.56-0.84)** |
| Arthritis (combined) | **1.31(1.03-1.66)** |
| Atlantoaxial instability | **10.31 (4.77-22.31)** |
| Autism | **5.06 (3.67-6.99)** |
| Chronic kidney disease | **2.59 (1.89-3.55)** |
| Coeliac disease | **10.12 (6.33-16.19)** |
| Congenital cardiac disease | **57.01 (46.05-70.56)** |
| Congenital gastrointestinal disease | **12.93 (8.06-20.75)** |
| Dementia | **24.01 (17.81-32.35)** |
| Dementia (≥30yrs at start of follow-up)” | **30.18 (21.10-43.17)** |
| Diabetes Mellitus (combined) | **2.13 (1.68-2.70)** |
| Diabetes Mellitus, Type 1^ | **3.01 (1.57-5.76)** |
| Diabetes Mellitus, Type 2^ | **1.56 (1.04-2.35)** |
| Duchenne muscular dystrophy | 2.77 (0.82-9.30) |
| Eczema | 0.97 (0.85-1.10) |
| Skin other | **2.29 (1.85-2.83)** |
| Epilepsy | **7.66 (6.26-9.38)** |
| Gastro-oesophageal reflux | **2.63 (2.25-3.07)** |
| Glaucoma | 1.66 (0.87-3.16) |
| Hearing impairment | **10.73 (8.98-12.82)** |
| Hyperthyroidism | **4.68 (3.11-7.05)** |
| Hypothyroidism | **14.75 (12.13-17.93)** |
| Inflammatory bowel disease | **2.39 (1.94-2.94)** |
| Iron deficiency anaemia | **1.84 (1.36-2.49)** |
| Ischaemic heart disease | **1.42 (1.07-1.89)** |
| Ischaemic heart disease (≥40yrs at start of follow-up)’ | **0.45 (0.28-0.74)** |
| Non-accidental injury/ maltreatment | 1.29 (0.89-1.86) |
| Schizophrenia | 1.59 (0.84-3.00) |
| Sleep disordered breathing | **7.30 (5.97-8.92)** |
| Stroke | **1.97 (1.36-2.85)** |
| Undescended testis | **3.34 (2.41-4.62)** |
| Vitamin D deficiency | **3.33 (2.01-5.52)** |
|  |  |

*Nb. Cases (individuals with DS) are matched with at least 4 matched controls (non-DS individuals) based on GP practice, practice level index of multiple deprivation, year of birth ± 1 year, sex and index date (the data at which a case is first labelled as having DS).*

*aOR = adjusted odds ratio; CI = 95% confidence intervals*

*Odds ratios are adjusted for ethnicity, smoking status and person years contributed.*

*Missing data: Ethnicity: DS=551, Control=6,068; Smoking status: DS=1,458 Controls=7,389*

*“DS N=2,163, Controls N=10,346*

*‘DS N=1,501, Controls N=6,596*

*^The prevalence of type 1 and type 2 diabetes (separately) is based on CPRD data only. It is not possible to differentiate between the subtypes of diabetes using HES data.*

*ADHD: Attention Deficit Hyperactivity Disorder*
