# Supplementary material for: Prevalence of multiple morbidities and cancers in individuals with Down syndrome: A matched descriptive study using linked electronic health record data
Source: PLoS One. 2026 Jun 3;21(6):e0349794. doi: 10.1371/journal.pone.0349794 (PMC13232805; doi:10.1371/journal.pone.0349794)
Supplement: S7 Table — (DOCX) [file pone.0349794.s009.docx]

**S7 Table: Primary analysis (adults & children): Further adjusted odds ratios (aOR) for the occurrence of cancers in the DS cohort v. matched controls.**

| **Cancer site** | **aOR (CI)**  **(95% CI </>1)** |
| --- | --- |
|  |  |
| Bladder | 0.62 (0.14-2.62) |
| Bone | 2.22 (0.25-19.73) |
| Brain/Central Nervous System | 1.05 (0.36-3.00) |
| Breast | 0.33 (0.11-1.07) |
| Cervix | **0.12 (0.03-0.49)** |
| Colorectal | 0.56 (0.27-1.15) |
| Gastro-oesophageal | - |
| Leukaemia | **12.68 (6.22-25.83)** |
| Liver/biliary | - |
| Lung | 0.14 (0.02-1.00) |
| Lymphoma | 0.65 (0.15-2.81) |
| Melanoma | - |
| Skin, non-melanoma | 0.49 (0.22-1.12) |
| Myeloma | - |
| Neuroblastoma | - |
| Ovarian | 1.55 (0.53-4.52) |
| Pancreas | 1.73 (0.47-6.35) |
| Prostate | 0.69 (0.21-2.22) |
| Renal | 0.58 (0.08-4.54) |
| Retinoblastoma | - |
| Testicular | **3.60 (1.15-11.21)** |
| Thyroid/parathyroid | 0.66 (0.08-5.16) |
| Uterus | 0.53 (0.22-1.33) |
| Wilms’ tumour | 0.68 (0.09-5.35) |
| Any of the cancers above | 0.83 (0.64-1.07) |

*Nb. Cases (individuals with DS) are matched with at least 4 matched controls (non-DS individuals) based on GP practice, practice level index of multiple deprivation, year of birth ± 1 year, sex and index date (the data at which a case is first labelled as having DS).*

*aOR = adjusted odds ratio; CI = 95% confidence intervals*

*Odds ratios are adjusted for ethnicity, smoking status and person years contributed.*

*Missing data: Ethnicity: DS=551, Control=6,068; Smoking status: DS=1,458 Controls=7,389*

*- = unable to calculate odd ratios due to absence of cancer in cases and/or controls, including after the inclusion of confounders in the model.*
