# Supplementary material for: Prevalence of multiple morbidities and cancers in individuals with Down syndrome: A matched descriptive study using linked electronic health record data
Source: PLoS One. 2026 Jun 3;21(6):e0349794. doi: 10.1371/journal.pone.0349794 (PMC13232805; doi:10.1371/journal.pone.0349794)
Supplement: S8 Table — (DOCX) [file pone.0349794.s010.docx]

**S8 Table: Subgroup-analysis (children only): Further adjusted odds ratios (aOR) for the occurrence of DS-associated morbidities and cancers in the DS cohort v. matched controls.**

| **Morbidity** | **aOR (CI)**  **(95% CI </>1)** |
| --- | --- |
|  |  |
| ADHD | 1.26 (0.74-2.13) |
| Anxiety/depression | **0.67 (0.43-1.03)** |
| Arthritis (combined) | **2.32 (1.15-4.69)** |
| Atlantoaxial instability | - |
| Autism | **5.17 (3.76-7.09)** |
| Chronic kidney disease | **3.69 (1.85-7.35)** |
| Coeliac disease | **11.18 (6.16-20.29)** |
| Congenital cardiac disease | **105.58 (81.18-137.33)** |
| Congenital gastrointestinal disease | **12.99 (7.93-21.28)** |
| Diabetes Mellitus (combined) | **3.93 (2.50-6.17)** |
| Diabetes Mellitus, Type 1^ | **3.35 (1.37-8.20)** |
| Duchenne muscular dystrophy | **8.15 (1.46-45.39)** |
| Eczema | **0.77 (0.66-0.89)** |
| Skin other | **1.91 (1.40-2.62)** |
| Epilepsy | **3.86 (2.84-5.23)** |
| Gastro-oesophageal reflux | **4.49 (3.73-5.42)** |
| Glaucoma | **14.95 (2.98-75.01)** |
| Hearing impairment | **14.07 (11.33-17.48)** |
| Hyperthyroidism | - |
| Hypothyroidism | **36.36 (24.66-53.62)** |
| Inflammatory bowel disease | **3.15 (2.45-4.03)** |
| Iron deficiency anaemia | **2.21 (1.46-3.34)** |
| Non-accidental injury/ maltreatment | **1.72 (1.21-2.45)** |
| Schizophrenia | 0.92 (0.11-7.96) |
| Sleep disordered breathing | **14.43 (11.29-18.44)** |
| Stroke | **12.28 (4.37-34.50)** |
| Undescended testis | **3.50 (2.55-4.81)** |
| Vitamin D deficiency | **3.30 (1.86-5.86)** |
| **Cancers** |  |
| Leukaemia | **63.72 (15.08-269.33)** |
| Lymphoma | 2.29 (0.58-9.02) |
| Neuroblastoma | - |
|  |  |

*Nb. Cases (individuals with DS) are matched with at least 4 matched controls (non-DS individuals) based on GP practice, practice level index of multiple deprivation, year of birth ± 1 year, sex and index date (the data at which a case is first labelled as having DS).*

*aOR = adjusted odds ratio; CI = 95% confidence intervals*

*Odds ratios are adjusted for ethnicity, and person years contributed.*

*Missing data: Ethnicity: DS=68, Control=1,205*

*^The prevalence of type 1 and type 2 diabetes (separately) is based on CPRD data only. It is not possible to differentiate between the subtypes of diabetes using HES data.*

*- = unable to calculate odd ratios due to absence of cancer in cases and/or controls..*

*ADHD: Attention Deficit Hyperactivity Disorder*
